# IL-15 signaling promotes adoptive effector T-cell survival and memory formation in irradiation-induced lymphopenia

**DOI:** 10.1186/s13578-016-0098-2

**Published:** 2016-05-06

**Authors:** Aizhang Xu, Kalpana Kalyanasundaram Bhanumathy, Jie Wu, Zhenmin Ye, Andrew Freywald, Scot C. Leary, Rongxiu Li, Jim Xiang

**Affiliations:** State Key Laboratory of Microbial Metabolism, Shanghai Jiao Tong University, Shanghai, China; School of Life Sciences and Biotechnology, Shanghai Jiao Tong University, Shanghai, China; Cancer Research Cluster, Saskatchewan Cancer Agency, Saskatoon, SK Canada; Departments of Oncology, University of Saskatchewan, HSB Room 4D30.1, 107 Wiggins Road, Saskatoon, SK S7N 5E5 Canada; Department of Pathology, University of Saskatchewan, Saskatoon, SK Canada; Department of Biochemistry, University of Saskatchewan, Saskatoon, SK Canada; Engineering Research Center of Cell & Therapeutic Antibody, School of Pharmacy, Ministry of Education, Shanghai Jiao Tong University, Shanghai, China

**Keywords:** Autophagy, Effector T-cells, IL-15, Irradiation, Lymphopenia, Mitochondrial biogenesis, T-cell memory, T-cell survival

## Abstract

**Background:**

Lymphopenia promotes naïve T-cell homeostatic proliferation and adoptive effector T-cell survival and memory formation. IL-7 plays a critical role in homeostatic proliferation, survival and memory formation of naïve T-cells in lymphopenia, and its underlying molecular mechanism has also been well studied. However, the mechanism for adoptively transferred effector T-cell survival and memory formation is not fully understood. Here, we transferred in vitro-activated transgenic OT-I CD8^+^ effector T-cells into irradiation (600 rads)-induced lymphopenic C57BL/6, IL-7 knockout (KO) and IL-15 KO mice, and investigated the survival and memory formation of transferred T-cells in lymphopenia.

**Results:**

We demonstrate that transferred T-cells prolong their survival and enhance their memory in lymphopenic mice, in a manner that depends on IL-15 signaling, but not IL-7. We determine that in vitro stimulation of naïve or effector T-cells with IL-7 and IL-15 reduces IL-7Rα, and increases and/or maintains IL-15Rβ expression, respectively. Consistent with these findings, the expression of IL-7Rα and IL-15Rβ is down- and up-regulated, respectively, in vivo on transferred T-cells in an early phase post T-cell transfer in lymphopenia. We further show that in vitro IL-15 restimulation-induced memory T-cells (compared to IL-2 restimulation-induced effector T-cells) and in vivo transferred T-cells in irradiated IL-15-sufficient C57BL/6 mice (compared to IL-15-deficient IL-15 KO mice) have increased mitochondrial content, but less NADH and lower mitochondrial potential (ΔΨm), and demonstrate greater phosphorylation of signal transducers and activators of transcription-5 (STAT5) and Unc-51-like kinase-1 (ULK1), and higher expression of B-cell leukemia/lymphoma-2 (Bcl2) and memory-, autophagy- and mitochondrial biogenesis-related molecules.

**Conclusion:**

Irradiation-induced lymphopenia promotes effector T-cell survival via IL-15 signaling the STAT5/Bcl2 pathway, enhances T-cell memory formation via IL-15 activation of the forkhead-box family of transcription factor (FOXO)/eomesodermin (Eomes) memory and ULK1/autophagy-related gene-7 (ATG7) autophagy pathways, and via IL-15 activation of the mitochondrial remodeling. Our data thus identify some important targets to consider when designing potent adoptive T-cell immunotherapies of cancer.

**Electronic supplementary material:**

The online version of this article (doi:10.1186/s13578-016-0098-2) contains supplementary material, which is available to authorized users.

## Background

Throughout life, T lymphocytes are maintained at fairly stable numbers by two distinct mechanisms, the activation-induced cell death (AICD) and the homeostasis. AICD results in the removal of a large number of active lymphocytes after massive antigen-induced clonal expansion [1]. The level of peripheral naive and memory T-cell pools is under tight homeostatic control, which is critical to the maintenance of both a polyclonal repertoire of naïve T-cells capable of responding to newly encountered pathogens, and a small population of antigen-experienced memory T (Tm) cells that provide protection against previously encountered pathogens [2].

T-cell homeostasis can be severely perturbed by factors such as ionizing radiation, which leads to large scale loss of naïve T or effector T (Te) cells due to apoptosis [3], and further results in transient or sustained lymphopenia [4]. During recovery from T-cell depletion, adoptive naïve T-cells are driven into cell division. This process known as homeostatic proliferation, replenishes the T lymphocyte pool [5], and leads to formation of CD44^+^CD62L^high^IL-7R^+^ memory-like Tm cells [6–8]. IL-7 is critical for homeostatic proliferation, survival and memory formation of adoptive naive T-cells in lymphopenia, and its underlying molecular mechanism has also been well studied [9, 10] However, the molecular cell-intrinsic mechanism for the prolonged survival and the enhanced memory formation of adoptive Te cells in lymphopenia has not been fully described.

In this study, we established an irradiation-induced lymphopenic animal model by irradiating C57BL/6 (B6) mice with 600 rads. Initially, we in vitro activated CD8^+^ T-cells purified from Ovalbumin (OVA)-specific T-cell receptor (TCR) transgenic OT-I mice with OVA I (OVA_257-264_) peptide and IL-2, and transferred the resulting Te cells into irradiation-induced lymphopenic B6, IL-7 knockout (KO) and IL-15 KO mice. T-cell survival and memory formation were quantified in these lymphopenic mice by flow cytometry, Western blotting and confocal microscopy analyses. We demonstrated that transferred Te cells exhibited prolonged survival and enhanced memory formation during lymphopenia, and that IL-15 signaling is central to the observed properties of transferred T-cells. Our experiments revealed that IL-15Rβ and IL-7Rα expression was up- and down-regulated, respectively, on transferred Te cells in an early phase post T-cell transfer in lymphopenia. Our work also showed that in vitro IL-15 restimulation-induced Tm cells (compared to IL-2 restimulation-induced effector T-cells) and in vivo transferred T-cells in irradiated IL-15-sufficient B6 mice (compared to IL-15 deficient IL-15 KO mice) had increased mitochondrial content, but less NADH and lower mitochondrial potential (ΔΨm), and demonstrated greater phosphorylation of signal transducers and activators of transcription-5 (STAT5) and Unc-51-like kinase-1 (ULK1), and higher expression of B-cell leukemia/lymphoma-2 (Bcl-2) and memory-, autophagy- and mitochondrial biogenesis-related molecules. Taken together, our data demonstrate that irradiation-induced lymphopenia promotes effector T-cells survival via IL-15 signaling the STAT5/Bcl-2 pathway, and enhances T-cell memory formation via IL-15 activation of the forkhead box (FOXO)/Eomesodermin (Eomes) memory and ULK1/autophagy-related gene 7 (Atg7) autophagy pathways and the mitochondrial remodeling.

## Results

### Irradiation-induced lymphopenia promotes naïve T-cell homeostatic proliferation

**Fig. 1:**
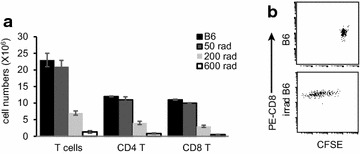
Irradiation-induced lymphopenia promotes homeostatic proliferation of naïve T-cells. **a** Irradiation induces lymphopenia in mice. B6 mice (n = 6) were irradiated at different doses. One day later, the total numbers of lymphocytes in spleen were measured. **b** Proliferation profiles of naïve CD8^+^ T-cells in lymphopenic mice and WT B6 mice. Naïve CD8^+^ T-cells labeled with CFSE were injected intravenously (i.v.) into irradiated (600 rads) B6 or WT B6 mice. Splenocytes were collected 6 days post T-cell transfer, stained with PE-ani-CD8 Ab, and then analyzed by flow cytometry. The CD8/CFSE double positive T-cells were gated for observation of divisions of CFSE-labeled T-cells. One representative experiment of three is shown

Irradiation can induce the large scale loss of naïve T-cells [11, 12], thereby leading to lymphopenia in irradiated mice [4]. Accordingly, one day after irradiating WT B6 mice with 50, 200 or 600 rads, we observed a dose-dependent depletion of naïve T-cells (Fig. 1a). To assess the potential for homeostatic proliferation of T-cells, we injected CFSE-labeled naïve CD8^+^ T-cells into B6 mice irradiated with 600 rads. Six days after T-cell transfer, we demonstrated that adoptive naïve T-cells are driven into homeostatic proliferation during recovery from T-cell depletion (Fig. 1b) [5], thus mice irradiated with 600 rads are lymphopenic.

### Lymphopenia promotes effector T-cell survival via up-regulation of anti-apoptotic Bcl-2 and down-regulation of pro-apoptotic Bax and Bcl-Xs

**Fig. 2:**
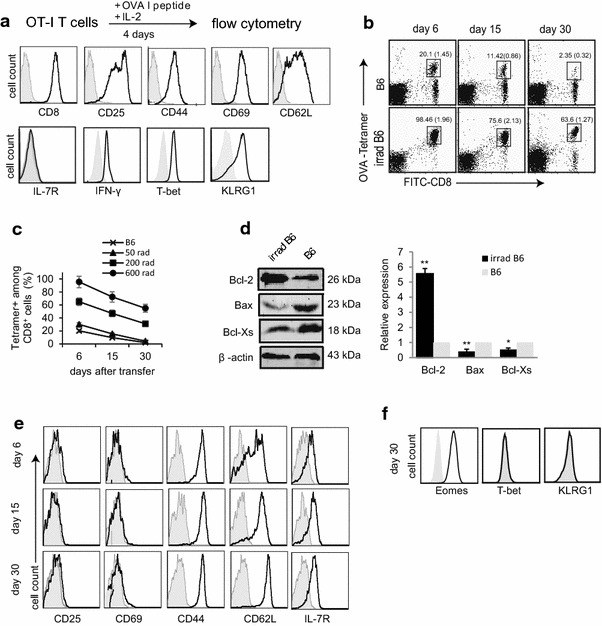
Irradiation-induced lymphopenia promotes survival and memory formation of transferred effector T-cells. **a** In vitro OVA-I peptide/IL-2 stimulated OT-I CD8^+^ T-cells were stained with various Abs (*solid lines*) and analyzed by flow cytometry. *Gray shaded histograms* represent isotype Ab controls. **b** Blood samples in WT B6 or irradiated (600 rads) B6 mice (n = 4) were collected and stained with PE-K^b^/OVA I-tetramer (OVA-tetramer), FITC-anti-CD8 Ab (FITC-CD8), and analyzed by flow cytometry at indicated times after T-cell transfer. The values represent the percentages of OVA-specific CD8^+^ T-cells in total CD8^+^ T-cell population. The values in parenthesis represent SD. **c** Kinetic assessment of transferred CD8^+^ T-cells in B6 mice irradiated with different doses by cytometry as described in (**b**). **d** Western blot analysis. Transferred T-cells were purified from WT B6 and irradiated (600 rads) B6 mice 6 days after T-cell transfer, and lysed for Western blot analysis. Relative expression represents the ratio of expression of each molecule in cells from irradiated B6 mice versus that in untreated control WT B6 mice. *p < 0.05, **p < 0.01. **e**, **f** Blood samples in irradiated (600 rads) B6 mice were collected and stained with PE-K^b^/OVAI-tetramer, FITC-anti-CD8 Ab and PE-Cy5-Abs specific for various molecules, and analyzed by flow cytometry at indicated times after T-cell transfer. The double positive (OVA-tetramer and CD8) T-cells were gated as in Fig. 2b for assessing the expression of CD25, CD69, CD44, CD62L and IL-7R (*solid lines*). Gray shaded histograms represent isotype Ab controls. One representative experiment of two is shown

To initiate the experiment, we activated OT-I CD8^+^ T-cells in vitro by treating them with OVA I peptide and IL-2 for 4 days, and then phenotypically characterized them by flow cytometry. We demonstrated that these in vitro-activated CD8^+^ T-cells lacked expression of memory marker IL-7R, but expressed active T-cell markers CD25 and CD69, the effector T (Te) cell markers IFN-γ and KLRG1, and the Te cell transcription factor T-bet (Fig. 2a), indicating that they are indeed bonafide Te-cells. To assess the lymphopenic effect on adoptive Te-cells in vivo, we transferred these Te-cells into B6 mice irradiated with different doses, and quantified the survival of OVA-specific CD8^+^ T-cells in peripheral blood by flow cytometry 6, 15 and 30 days post T-cell transfer. We found that the survival of transferred CD8^+^ T-cells in irradiated B6 mice was significantly prolonged, in a manner that depends on irradiation doses (50, 200 and 600 rads), compared to WT B6 mice, in which the transferred T-cells declined dramatically (Fig. 2b, c). We, therefore, selected an irradiation dose of 600 rads for inducing lymphopenia in this study. To assess the mechanism for prolonged survival, we measured expression of apoptosis-associated molecules by Western blot analysis. We demonstrated that there was fivefold increase in the abundance of the anti-apoptotic factor Bcl-2, and twofold reduction in the steady-state levels of the pro-apoptotic factors Bcl-Xs and Bax in transferred CD8^+^ T-cells of irradiated B6 mice, compared to WT B6 mice (Fig. 2d).

### Lymphopenia also enhances T-cell memory formation

We then addressed whether T-cells transferred into WT B6 and irradiated B6 mice exhibited any differences with respect to memory phenotype by flow cytometry. Flow cytometry analyses demonstrated that the transferred T-cells gradually up-regulated expression of memory marker IL-7R, while rapidly down-regulating active T-cell markers CD25 and CD69 at day 6 and 15 post T-cell transfer (Fig. 2e). After 30 days, when transferred T-cells become long-term memory T (Tm) cells [13], they did express Tm cell markers CD44, IL-7R and CD62L as well as Tm cell transcription factor Eomes, but lacked expression of active and effector T-cell markers CD25, CD69, KLRG1 and T-bet in irradiated B6 mice (Fig. 2e, f) and in WT B6 mice (data not shown). In addition, detected Tm cells (64 %) in irradiated B6 mice were much higher than those (2 %) in WT B6 mice (Fig. 2b), indicating that irradiation-induced lymphopenia promotes Tm cell formation.

### IL-15 plays an important role in promoting T-cell survival and memory in lymphopenia

**Fig. 3:**
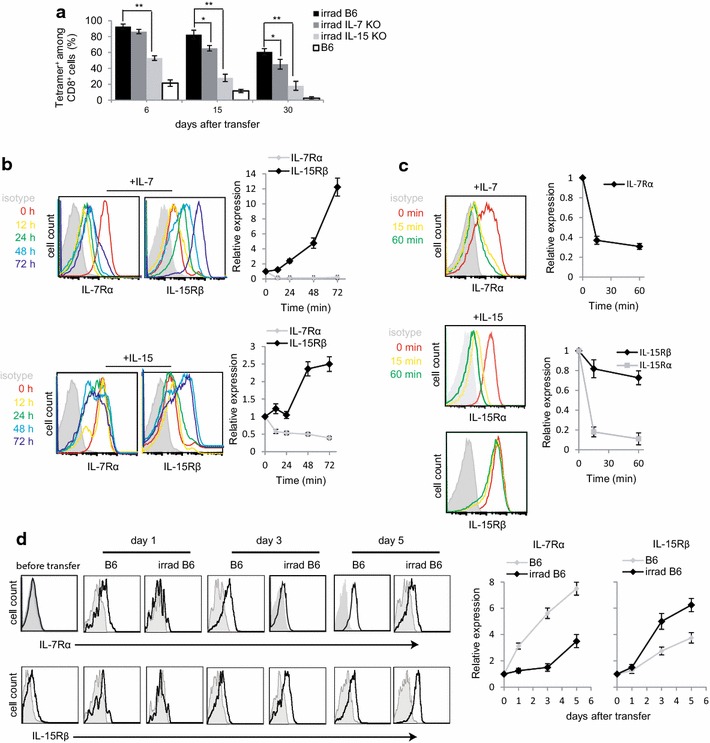
IL-7 and IL-15 distinctly modulate expression of IL-7Rα and IL-15Rβ. **a** Mouse blood samples were analyzed by flow cytometry at indicated times after T-cell transfer. The percentages of transferred OT-I CD8^+^ T-cells in the total CD8^+^ T-cell population were assessed. **b** Naïve OT-I CD8^+^ T-cells cultured in medium containing OVA I peptide and IL-7 or IL-15 (10 ng/ml) were analyzed by flow cytometry at indicated times. Relative expression represents the ratio of mean fluorescence intensity (MFI) for expression of each molecule at indicated time points versus that at the beginning (0 h) in naïve T-cells. **c** Naïve OT-I CD8^+^ cells were activated in complete medium containing OVA I peptide and IL-2 for 3 days, followed by starvation in complete medium without cytokine for 5 h. The resulting cells were further in vitro re-stimulated with IL-7 or IL-15 (10 ng/ml). The re-stimulated T-cells were then analyzed by flow cytometry. Relative expression represents the ratio of MFI for expression of each molecule at indicated time points versus that at the beginning (0 min) in effector T-cells. **d** Blood samples (n = 4) analyzed by flow cytometry at indicated days. The double positive (OVA-tetramer and CD8) T-cells were gated for further assessing the expression of IL-7Rα and IL-15Rβ (*solid lines*). *Gray shaded histograms* represent isotype Ab controls. Relative expression represents the ratio of MFI for the expression of each molecule at indicated time points in transferred effector T-cells post T-cell transfer versus that in effector T-cells before T-cell transfer. *p < 0.05, *p < 0.01. One representative experiment of three is shown

The prolonged T-cell survival in lymphopenic mice may arise from microenvironment homeostasis, related to decreased competition for T-cell homeostatic cytokines such as IL-7 and IL-15 [14]. To address this possibility, we transferred the in vitro-activated OT-I CD8^+^ Te cells into irradiated (600 rads) WT B6, IL-7 KO and IL-15KO mice, followed by monitoring kinetics of their survival and memory formation. Consistent with our previous results (Fig. 2b), we showed that most (91 and 80 %) of T-cells transferred into irradiated WT B6 mice remained viable at day 6 and 15, and eventually became Tm-cells (60 %) at day 30 post T-cell transfer (Fig. 3a). T-cell survival and memory formation were only slightly affected by depletion of IL-7 in irradiated IL-7 KO mice (Fig. 3a). In contrast, only 53 and 28 % of transferred T-cells survived at day 6 and 15, and only 18 % of transferred T-cells became Tm cells at day 30 post T-cell transfer in irradiated IL-15 KO mice (Fig. 3a). It was previously reported that IL-15 plays an important role in KLRG1^hi^IL-7Rα^lo^ effector CTL survival and memory formation in WT mice [15]. Collectively, our findings indicate that IL-15 signaling is also crucial for prolonged T-cell survival and enhanced T-cell memory formation in lymphopenic mice.

### Transferred Te cells up-regulate IL-15Rβ, but down-regulate IL-7Rα expression early on post T-cell transfer in lymphopenia

To assess a potential mechanism by which IL-15 contributes to the prolonged survival and enhanced memory of transferred T-cells, we first isolated naïve CD8^+^ T-cells from transgenic OT-I mice, and then measured IL-7Rα and IL-15Rβ expression in response to in vitro stimulation with OVA I peptide and IL-7 or IL-15. We found that IL-7 stimulation dramatically down-regulated IL-7Rα, but gradually up-regulated IL-15Rβ expression (Fig. 3b). In contrast, treatment of IL-15 gradually up-regulated IL-15Rβ, but dramatically down-regulated IL-7Rα expression in T-cells (Fig. 3b). We next assessed the stimulatory effect of IL-7 and IL-15 on Te cells, thus mimicking the situation of our study of the in vivo effect of IL-15 signaling on transferred Te cells in lymphopenia. In vitro OVAI/IL-2-stimulated OT-I Te cells were further cultured in medium containing IL-7 or IL-15, and the expression of IL-7Rα and IL-15Rβ was quantified by flow cytometry. Interestingly, we found that co-culture with IL-7 down-regulated the expression of IL-7Rα, whereas expression of IL-15Rβ in re-stimulated T-cells was relatively unchanged upon treatment of IL-15 (Fig. 3c). To further investigate the differential contribution of IL-15 relative to IL-7 with respect to the prolonged survival and enhanced memory of transferred T-cells in lymphopenia, we isolated naïve CD8^+^ T-cells from transgenic OT-I mice, stimulated them with OVA I peptide and IL-2 for 4 days, transferred them into WT B6 and irradiated B6 mice, and quantified changes in the expression of IL-7Rα and IL-15Rβ over time. We found that transferred T-cells did not express any IL-7Rα and IL-15Rβ on day 1 post T-cell transfer in both WT and irradiated B6 mice (Fig. 3d). Interestingly, however, transferred T-cells in irradiated B6 mice expressed less IL-7Rα, but more IL-15Rβ, 3 days post T-cell transfer, when compared to WT B6 mice (Fig. 3d). In addition, we found that IL-15Rα which was downregulated in IL-15 treated cells in vitro (Fig. 3c) was upregulated in the transferred effector T-cells in lymphopenic mice compared to WT mice (Additional file 1: Figure S1a). The upregulation may result from the released IL-15Rα from DCs or monocytes after irradiation. We also found that the expression of IL-15Rα increased in effector T-cells co-cultured with irradiated DC in vitro (Additional file 1: Figure S1b).

### In vitro IL-15-restimulated T-cells become memory-like Tm cells with prolonged survival and enhanced memory formation following their transfer into B6 mice

**Fig. 4:**
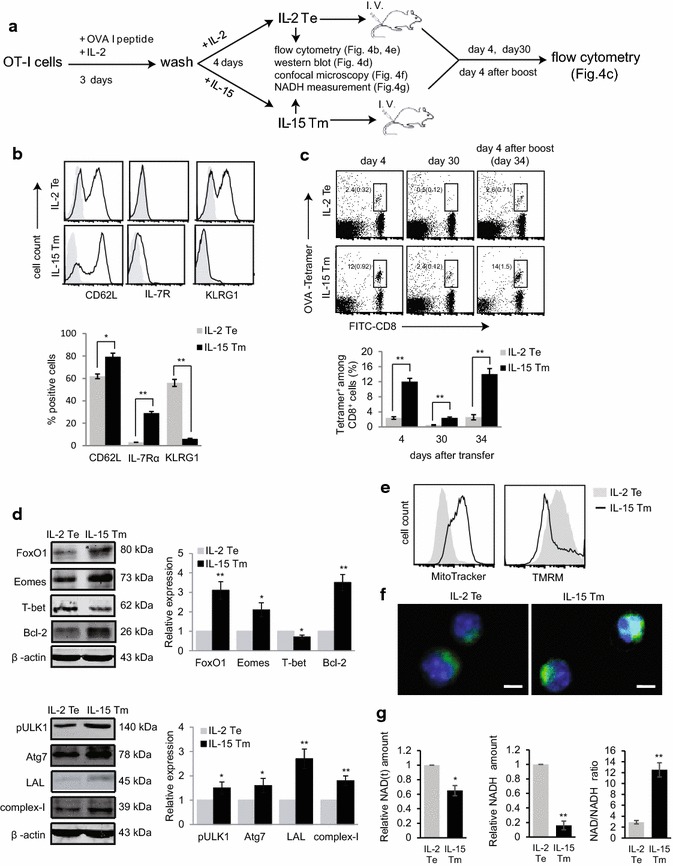
In vitro IL-15 signaling converts effector T-cells to memory T-cells with prolonged survival and enhanced memory. **a** Schematic diagram of the experiment design. **b** OVAI/IL-2-activated OT-I CD8^+^ T-cells were restimulated with IL-2 or IL-15, and became IL-2 Te or IL-15 Tm cells. They were stained with Abs, and analyzed by flow cytometry (*solid lines*). *Gray shaded histograms* represent isotype Ab controls. The percentages of CD62L, IL-7Rα and KLRG1 positive cells were shown. **c** IL-2 Te and IL-15 Tm cells (20 × 10^6^) were adoptively transferred into B6 mice (n = 4), and blood samples were collected at day 4, day 30, and day 34 [4 days post DC_OVA_ (1 × 10^6^) boost for recall responses] post T-cell transfer, and analyzed by flow cytometry. The value in each panel represents the percentage of OVA-specific (OVA-tetramer positive) CD8^+^ T-cells within the total CD8^+^ T-cell population. **d** Western blot analysis of IL-2 Te and IL-15 Tm cell lysates using Abs specific for FOXO1, Eomes, T-bet, Bcl-2, pULK1, Atg7, complex-I. Relative expression represents the ratio of the expression of each molecule in IL-15 Tm cells versus that in IL-2 Te cells. **e** IL-2 Te (*gray shaded histograms*) and IL-15 Tm cells (*solid lines*) were stained with MitoTracker and TMRM, and analyzed by flow cytometry for measurement of mitochondrial mass and mitochondrial membrane potential, respectively. **f** Confocal images show IL-2 Te and IL-15 Tm cells stained with Mitotracker (*green*) and Hoechst (*blue*); scale bars represent 5 μm. **g** NADH amount in IL-2 Te and IL-15 Tm cells. Relative amount of NAD(t) (total NAD^+^ and NADH) and NADH and NAD/NADH ratio measured in IL-15 Tm cells and in IL-2 Te cells are shown. *p < 0.05, **p < 0.01. One representative experiment of three is shown

We next sought to assess the effect of in vitro IL-15 stimulation on T-cell survival and memory formation in vivo, we first isolated naïve CD8^+^ T-cells from transgenic OT-I mice, and stimulated them with OVA I peptide and IL-2 for 3 days, and then co-cultured them with IL-2 or IL-15 for 4 days [16] prior to intravenous injection of them into B6 mice (Fig. 4a). We found that unlike the IL-2 re-stimulation-induced effector T (short term: IL-2 Te) cells, which expressed KLRG1, IL-15 stimulated T-cells did not, and instead up-regulated expression of the memory T-cell markers IL-7R and CD62L (Fig. 4b). These findings indicate that in vitro IL-15 stimulated T-cells became IL-7R^+^CD62L^high^KLRG1^−^ memory-like Tm (short term: IL-15 Tm) cells [16]. CD8^+^ Tm cells are characterized by their capacity for prolonged survival and vigorous recall responses upon antigen re-encounter [17]. We transferred IL-2 Te cells and IL-15 Tm cells into WT B6 mice to directly assess their memory T-cell characteristics (Fig. 4a). Compared to IL-2 Te cells, IL-15 Tm cells exhibited prolonged survival and enhanced memory formation (Fig. 4c). To confirm these findings, we boosted mice with DC_OVA_, and measured recall responses by flow cytometry 4 days after the boost. Indeed, we found roughly a sixfold increase of OVA-specific T-cells (Fig. 4c), indicating that these Tm cells are all functional.

### In vitro IL-15 signaling converts Te cells to Tm cells by altering the expression of key regulators of gene expression and autophagy and the number and functional properties of mitochondria

To dissect molecular pathways regulating in vitro IL-15 restimulation-induced prolonged T-cell survival and enhanced T-cell memory formation, we measured the expression of molecules controlling T-cell apoptosis, memory and autophagy, and the content of mitochondria (Fig. 4a). Compared to IL-2 Te cells, IL-15 Tm cells up-regulated the expression of anti-apoptotic factor Bcl-2, increased the expression of the memory T-cell transcription factors FoxO1 and Eomes, the autophagy-related molecules Atg7 and lysosomal acid lipase (LAL), and the 39 kDa structural subunit of complex-I of the mitochondrial electron transport chain (ETC) and enhanced the phosphorylation of ULK1 (pULK1) (Fig. 4d). Flow cytometry with MitoTracker Green, which binds to mitochondrial lipid membranes independent of their potential [18], demonstrated that the content of mitochondria was higher in IL-15 Tm cells than in IL-2 Te cells (Fig. 4e, f). To complement these analyses, we also measured mitochondrial membrane potential (ΔΨm) represented by tetramethylrhodamine ester (TMRM) staining, and found that it was lower in IL-15 Tm cells (Fig. 4e). Finally because NADH is generated by the tricarboxylic acid (TCA) cycle and donates its electrons to complex-I, a core component of the mitochondrial oxidative phosphorylation (OXPHOS) system responsible for aerobic ATP production [19], we quantified the amount of NAD(H) (total NAD + and NADH) (Fig. 4a). We observed that IL-15 Tm cells had less NADH, as demonstrated by a lower amount of total NAD(H) and a higher NAD/NADH ratio than IL-2 Te cells (Fig. 4g). Collectively, our data indicate that in vitro IL-15 signaling is able to convert Te cells to Tm-cells by up-regulating the expression of Bcl-2, FOXO1, Eomes, Atg7, ETC-protein complex-1 and LAL, increasing phosphorylation of ULK1, and enhancing mitochondrial biogenesis.

In vivo transferred T-cells up-regulate expression of Bcl-2, FoxO1, Eomes and Atg7, enhance the phosphorylation of pSTAT5 and ULK1 and activate the mitochondrial biogenesis via IL-15 signaling in lymphopenia.

**Fig. 5:**
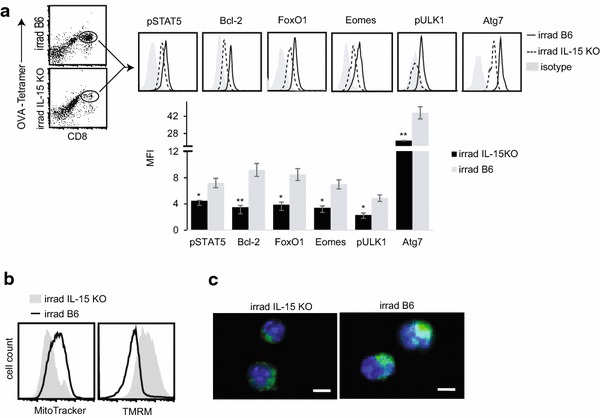
In vivo IL-15 signaling converts transferred effector T-cells to memory-like Tm cells in lymphopenia. **a** Irradiated (600 rads) B6 and IL-15 KO mice (n = 4) were transferred with OT-I effector T-cells (20 × 10^6^). Six days after transfer, splenocytes were stained with OVA-tetramer, PE-CY5-anti-CD8 Ab and FITC-Abs specific for pSTAT5, Bcl-2, FOXO1, Eomes, pULK1 and Atg7, and analyzed by flow cytometry. The double positive (OVA-tetramer and CD8) T-cells were gated for assessing the expression of various molecules in irradiated B6 mice (*solid lines*) and in irradiated IL-15 KO mice (*dotted lines*). *Gray shaded histograms* represent isotype Ab controls. MFI for expression of each molecule is shown. *p < 0.05, **p < 0.01. **b** Transferred OVA-specific T-cells in irradiated B6 and IL-15 KO mice were purified from spleens, 6 days post T-cell transfer, with PE-conjugated H-2 K^b^/OVA_257–264_ tetramer and anti-PE-microbeads, stained with MitoTracker and TMRM, and then analyzed by flow cytometry (irradiated B6 mice, *solid lines*; IL-15 KO mice, *gray shaded histograms*). **c** Confocal images show transferred T-cells stained with Mitotracker (*green*) and Hoechst (*blue*); *scale bars* represent 5 μm. One representative experiment of three is shown

To assess whether the above conclusion derived from our in vitro studies is relevant to the observed prolonged T-cell survival and enhanced T-cell memory in the lymphopenia model, we analyzed the expression of molecules controlling T-cell apoptosis, memory and autophagy in transferred T-cells. We demonstrated that transferred T-cells with prolonged survival up-regulated the expression of Bcl-2, FOXO1, Eomes and Atg7 to a greater extent, and enhanced the phosphorylation of STAT5 and ULK1 in irradiated B6 mice expressing IL-15 (Fig. 5a). In addition, we showed that transferred T-cells in irradiated B6 mice had a greater number of mitochondria (Fig. 5b) that were more tightly packed (Fig. 5c), and had lower mitochondrial membrane potential (ΔΨm) (Fig. 5b), when compared to those purified from irradiated IL-15 deficient IL-15 KO mice. These findings provide further evidence that IL-15 signaling is a critical regulator essential for T-cell biology in lymphopenia.

## Discussion

In this study, we generated an irradiation-induced lymphopenic mouse model. To assess a potential effect of lymphopenia on Te cells, we transferred active OT-I CD8^+^ T-cells into WT B6 or irradiated B6, and demonstrate that irradiation (600 rads)-induced lymphopenia promotes Te cell survival and Tm cell formation. To assess whether IL-7 or IL-15 plays a major role in these effects, we transferred active OT-I CD8^+^ T-cells into irradiated B6, IL-7 KO and IL-15 KO mice. We demonstrate that transferred T-cells gradually up-regulate IL-7Rα and IL-15Rβ expression in both WT and irradiated B6 mice, and the prolonged survival and enhanced memory are mildly reduced in irradiated IL-7 deficient IL-7 KO mice, consistent with a previous report [20], but dramatically decreased in irradiated IL-15 deficient IL-15 KO mice. Collectively, our results indicate that IL-15 is important for the survival and memory formation of transferred Te cells in lymphopenic mice, in comparison to previously reported IL-7 that plays a critical role in homeostatic proliferation, survival and memory formation of adoptive naive T-cells in lymphopenia [9, 10].

To begin to address why IL-15 figures more prominently than IL-7 in T-cell reprogramming in lymphopenia though both of them are of similar functional effects [21], we first assessed changes in expression of IL-7Rα and IL-15Rβ on naïve T-cells or Te cells following in vitro stimulation with IL-7 or IL-15. Consistent with a previous report [22], we found that IL-7 treatment down-regulates IL-7Rα expression, while co-culture with IL-15 either up-regulates IL-15Rβ expression on stimulated “naïve” T-cells or maintains IL-15Rβ expression on IL-15-restimulated Te cells. We next extended these in vitro analyses, and reveal, for the first time, a distinct in vivo modulation of IL-7Rα and IL-15Rβ expression on transferred Te cells in lymphopenia. We determine that IL-7Rα expression is transitionally down-regulated, whereas IL-15R expression is up-regulated in the early phase post T-cell transfer in lymphopenic mice. It is well-known that irradiation removes the sinks for homeostasis cytokines such as IL-7 and IL-15 [23] leading to IL-7 and IL-15 increase in lymphopenia [24, 25], and induces radiation-sensitive hematopoietic cell apoptosis, leading to releasing its IL-15Rα and subsequent formation of IL-15/IL-15Rα complexes [26, 27]. As such, IL-15 in the form of IL-15/IL-15Rα complexes thus becomes abundant in the host after irradiation. It is therefore tempting to speculate that transferred T-cells receive pro-survival cytokine stimulation mainly derive from IL-15, but not IL-7, in early phase post T-cell transfer, because IL-15Rβ expression is up-regulated and soluble IL-15/IL-15Rα complexes provide very sensitive and potent IL-15 signaling via binding of IL-15/IL-15Rα complexes to IL-15Rβ on transferred T-cells [27, 28]. This would provide a plausible mechanistic explanation for a dominant role for IL-15 in prolonged T-cell survival and enhanced Tm cell formation of transferred Te cells in lymphopenia.

T-cell apoptosis is to a large extent governed by members of the Bcl-2 family [29] composed of both pro- and anti-apoptotic molecules [30]. Over-expression of anti-apoptotic Bcl-2 inhibits Te cell death [31], whereas over-expression of pro-apoptotic Bcl-Xs and Bax accelerate T-cell apoptosis [32, 33]. It has been demonstrated that phosphorylation of STAT5 is required to maintain Bcl-2 expression in Te cells for their survival [34]. In this study, we show that transferred T-cells with prolonged survival up-regulate expression of Bcl-2, but down-regulate Bax and Bcl-Xs in irradiated B6 mice. We also demonstrate that transferred T-cells with prolonged survival in irradiated IL-15 sufficient B6 mice increase phosphorylation of STAT5 and up-regulate expression of Bcl-2, when compared to those in irradiated IL-15 deficient mice, further arguing that irradiation-induced lymphopenia promotes T-cell survival via IL-15 stimulation of the STAT5/Bcl-2 pathway.

The FoxO1 transcription factor promotes T-cell survival [35] and memory [36] via up-regulating memory T-cell transcription factor Eomes [37], and inhibits the memory target of rapamycin complex-1 (mTORC1), leading to down-regulation of the Te cell transcription factor T-bet [37]. To assess the molecular pathway responsible for enhanced Tm cell formation in lymphopenia, we measured the expression of FOXO1 and Eomes, and demonstrate that in vitro IL-15 Tm cells (compared to IL-2 Te cells) and in vivo transferred T-cells in IL-15 sufficient B6 mice (compared to those in IL-15 deficient mice) indeed up-regulate the expression of FOXO1 and Eomes in an IL-15 dependent manner, indicating that lymphopenia promotes Tm-cell formation via IL-15 activation of the FOXO/Eomes memory pathway for memory programming.

CD8^+^ Te cells depend on glycolysis to provide energy for proliferation and effector functions, whereas Tm cells depend on lipid oxidation for maintenance of their survival and memory phenotype [38]. The catabolic activity of autophagy is essential for growth, proliferation and homeostasis of these cells [39]. In the immune system, autophagy-related molecules such as the serine/threonine kinase ULK1, autophagy-initiating kinase Atg7 and LAL have been found to be essential for mitochondrial health, effector T-cell viability and CD8^+^ Tm cell formation [40–43]. In this study, we demonstrate a key role both in vitro and in vivo for IL-15 in enhancing the phosphorylation of ULK1 and up-regulating the expression of Atg7 and LAL, indicating that lymphopenia promotes Tm cell formation via IL-15 dependent activation of the ULK1/Atg7 autophagy pathway for maintenance of their memory metabolism.

Naïve T-cells mainly use mitochondrial oxidative phosphorylation (OXPHOS) and fatty acid oxidation (FAO) for energy in the form of ATP [44]. After antigen encounter, T-cells shift to glycolytic metabolism to sustain proliferation and effector functions [45]. During Tm cell development, T-cells down-regulate glycolytic pathways and revert back to catabolic metabolism, by using mainly OXPHOS and FAO. They up-regulate mitochondrial biogenesis, resulting in an increased mitochondrial spare respiratory capacity (SRC) to promote long-term survival [46]. IL-15 is known to support SRC and promotes the use of FAO for energy via enhanced mitochondrial biogenesis, leading to Tm cell development [47]. In this study, we find that Tm-cells stimulated with IL-15 in vitro and T-cells transferred into irradiated B6 mice expressing IL-15 have higher mitochondrial contents and increased abundance of a catalytic subunit of ETC protein complex-I important for Tm cell function [48]. NADH is generated by the tricarboxylic acid (TCA) cycle and donates electrons to complex-I as part of OXPHOS [19]. We show that IL-15 Tm cells have less NADH, but a higher NAD/NADH ratio than IL-2 Te cells, suggesting that IL-15 Tm cells preferentially use OXPHOS to generate energy. Mitochondrial membrane potential (ΔΨm) is a metabolic parameter that represents the proton motive force (Δp) across the inner membrane, and is an essential facet of aerobic ATP synthesis [38]. Interestingly, we also show that Tm cells stimulated with IL-15 in vitro and T-cells transferred into irradiated B6 mice expressing IL-15 have lower ΔΨm, a newly discovered metabolic signature of decreased glycolysis and increased SRC for prolonged T-cell survival and memory formation [49].

Adoptive T-cell immunotherapy for cancer, which was based upon the adoptive transfer of in vitro active tumor-specific CD8^+^ T-cells into cancer patients, previously achieved only some degree of success [50, 51], mostly due to their very short lifespan in vivo [52, 53]. The recent development of adoptive T-cell therapy using T-cells transfected with chimeric antigen receptor containing an intra-cellular signaling domain has greatly improved survival of transferred tumor-specific T-cells in patients [54]. Interestingly, the lymphopenic conditions also significantly enhanced the efficacy of adoptively transferred tumor-specific CD8^+^ T-cells by prolonging their survival leading to memory-like Tm cells [23, 55, 56], superior to in vitro expanded Te cells in controlling tumor growth [57]. In addition, the exposure to IL-15 also promotes an optimal response of CD8^+^ Tm cells with rapid division following antigen re-counter, and enhanced protective capacity against tumor cells [58].

## Conclusion

Taken together, our data demonstrate that irradiation-induced lymphopenia promotes adoptive Te cell survival via IL-15 signaling the STAT5/Bcl-2 pathway, and enhances T-cell memory formation via IL-15 activation of the FOXO/Eomes pathway for memory programming and the ULK1/Atg7 autophagy pathway and via IL-15 activation of the mitochondrial biogenesis for maintenance of their memory metabolism. We believe that this novel information should have an important impact on designing potent adoptive T-cell immunotherapies of treating cancer.

## Methods

### Antibodies and animals

The fluorescein isothiocyanate (FITC) anti-mouse Bcl-2 antibody (Ab) was purchased from BD-Biosciences (Mississauga, Ontario, Canada). Mouse IL-15Rα biotinylated Ab was purchased from R&D system (Minneapolis, MN). The biotin-labeled Abs for IL-15Rβ (5H4) and KLRG1 (2F1), the phycoerythrin (PE)-CY5-labeled Ab for CD8 (53-6.7), PE-labeled Abs for Eomes (Dan11mag), IFN-γ (XMG1.2) and T-bet (eBio4B10), and PE-CY5 labelled streptavidin were purchased from eBiosciences (San Diego, CA). The biotin-labeled Abs for CD25 (7D4), CD44 (IM7), CD62L (MEL-14), CD69 (H1.2F3), Ly6C (HK1.4) and IL-7Rα (SB/199) were purchased from Biolegend (San Diego, CA). Rabbit Ab for Atg7 and the mitochondrial complex-I structural subunit NDUFA9 (20C11B11B11) were purchased from Abcam (Cambridge, UK). Rabbit Abs for Bcl-2, Bax, Bcl-Xs, Eomes, T-bet and β-actin, mouse Ab for LAL (9G7F12) were purchased from Santa Cruz Biotechnology (Dallas, Texas, USA). Rabbit Abs for pULK1 (p-S_467_), FoxO1 and pSTAT5 (p-Y_694_) were purchased from Cell Signaling Technology (Beverly, MA). PE-labeled H-2 K^b^/OVA_257–264_ tetramer and FITC-labeled anti-CD8 (KT15) Ab were obtained from Beckman Coulter (San Diego, CA). Ovalbumin (OVA) I peptide (OVA_257–264_, SIINFEKL) specific for H-2 K^b^ was synthesized by Multiple Peptide Systems (San Diego, CA). The goat anti-rabbit or mouse IRDye^R^800CW Ab was purchased from LI-COR Bioscience (Lincoln, Nebraska). Female wild-type (WT) B6 mice, and OVA-specific TCR transgenic OT-I and IL-7 knockout (IL-7 KO) mice on B6 background were purchased from Jackson Laboratory (Bar Harbor, MA). IL-15 knockout (IL-15 KO) mice on B6 background were obtained from Taconic Biosciences (Rensselaer, NY). All mice were housed in the animal facility at the Health Sciences Building, and treated according to Animal Care Committee guidelines of University of Saskatchewan.

### Activation of CD8^+^ T-cell in vitro

Naive CD8^+^ T-cells were isolated from the spleens of transgenic OT-I mice, enriched by passage through nylon wool columns (C&A Scientific, Manassas, VA), and then fractionated by negative selection using anti-mouse CD4 (L3T4) paramagnetic beads (DYNAL Inc., Lake Success, NY) according to the manufacturer’s protocols to yield T-cell populations that were ~95 % CD8^+^/Vα2 Vβ5. To generate active T-cells, CD8^+^ T-cells purified from OT-I mice were activated in complete RPMI medium containing 100 U/ml IL-2, 50 µM 2-mercaptoethanol (2-ME), and 0.1 nM OVA-I peptide (SIINFEKL) for 3 or 4 days [47]. A portion of these active OT-I mouse CD8^+^ T-cells were intravenously (i.v.) transferred into irradiation-induced lymphopenic mice. The remainders were washed with PBS, and then further cultured in complete RPMI medium containing either IL-2 (100 U/ml) or IL-15 (10 ng/ml) (Peprotech, Rocky Hill, NJ) for an additional 4 days to generate in vitro IL-2-restimulated and in vitro IL-15-restimulatied T-cells. All cytokine-stimulated OT-I mouse CD8^+^ T-cells were analyzed by flow cytometry or lysed in RIPA lysing buffer (Thermo Scientific, Waltham, MA) containing both Halt Protease and Halt Phosphatase Inhibitor Cocktails (Thermo Scientific) for Western blot analysis.

### In vivo CD8^+^ T-cell survival and memory information

Wild-type and irradiated (600 rads) B6, IL-7 KO and IL-15 KO mice were i.v. transferred with the above in vitro activated CD8^+^ T-cells (20 × 10^6^ cells per mouse). Tetramer staining assay was performed to examine the presence of OVA-specific CD8^+^ T-cells in mouse peripheral blood at day 6, 15, 30 post T-cell transfer. The tail blood samples were stained with PE-H-2 K^b^/OVA_257–264_ tetramer, FITC-anti-CD8, and some PE-CY5-antibodies, respectively, and then analyzed by flow cytometry.

### Dendritic cells preparation

Bone marrow-derived dendritic cells (DCs) were obtained by culturing bone marrow cells of WT B6 in culture medium containing granulocyte monocyte colony-stimulating factor (GM-CSF) (20 ng/ml) and IL-4 (20 ng/ml) (Peprotech) for 6 days as previously described [59]. DCs were pulsed with OVA (0.5 mg/ml) for overnight and termed DC_OVA_.

### Western-blotting

Transferred OVA-specific CD8^+^ T-cells in mice were purified, 6 days post T-cell transfer, with PE-conjugated H-2 K^b^/OVA_257–264_ tetramer and anti-PE-microbeads (Miltenyi Biotech, Auburn, CA) [60] and lysed in ice-cold RIPA lysing buffer. Equal amounts of cell lysates were loaded onto 12 % acrylamide gels (SDS-PAGE) and transferred onto nitrocellulose membrane. The membrane was blocked with OYSSEY blocking buffer (LI-COR Bioscience), and immunoblotted with various antibodies polyclonal rabbit antibodies specific for Bax, Bcl-Xs and Bcl-2 at 4 °C for overnight. The membrane was detected with goat anti-rabbit IRDye^R^800CW and scanned under ODYSSEY instrument according to manufacturer’s instruction (LI-COR Bioscience). Band intensities were analyzed by using Image J software.

### Flow cytometry for intracellular markers

WT B6 mice and irradiated (600 rads) B6, IL-7 KO and IL-15 KO mice were i.v. transferred with the above in vitro activated OT-I CD8^+^ T-cells (20 × 10^6^ cells per mouse). Six days after T-cell transfer, mice tail blood were collected and stained with PE-K^b^/OVAI-tetramer and PE-Cy5-conjugated anti-CD8 Ab. After permeabilization using Cytoperm™ Permeabilization Buffer (BD-Biosciences), cells were stained with FITC-labeled anti-pSTAT5, anti-Bcl-2, anti-Eomes, anti-FoxO1 and anti-Atg7 Abs, and then analyzed by flow cytometry.

### Mitochondrial assays

The mitochondria mass and mitochondrial membrane potential (ΔΨm) were measured by MitoTracker Green (Life Technologies) and tetramethylrhodamine ester (TMRM), respectively, as previously described [61]. Briefly, in vitro IL-2-restimulated and IL-15-restimulated T-cells were stained with MitoTracker Green at 50 nM or TMRM at 10 µM for 15 min at 37 °C, respectively, according to the manufacturer’s manual. NAD(H) measurements were performed with the NAD^+^ and NADH Quantification kit (BioVision, Milpitas, CA), according to the manufacturer’s manual.

### Confocal microscopy

In vitro IL-2-restimulated and IL-15-restimulated T-cells or transferred OVA-specific CD8^+^ T-cells in irradiated B6 or IL-15 KO mice, which were purified, six days post T-cell transfer, with PE-conjugated H-2 K^b^/OVA_257–264_ tetramer and anti-PE-microbeads (Miltenyi Biotech), were stained with MitoTracker Green and TMRM, washed three times with PBS, and then incubated with 5 μg/ml of Hoechst 33342 (Life Technologies) for 20 min at room temperature. Fluorescence images were acquired using the acquisition software FV1000 Version 1 (FV10-ASW) on an inverted IX81 confocal microscope (FV1000) (Olympus, Shinjuku, Tokyo, Japan).

### Statistical analysis

Unless stated otherwise, data are expressed as mean (with SD). Statistical analyses were performed using the Mann–Whitney *U* test for comparison of variables from different groups in animal studies or the Student *t* test for comparison of variables from different groups in all other studies. Probability values of *p* > 0.05, *p* < 0.05 and *p* < 0.01 are considered statistically not significant, significant and very significant, respectively.

## Additional file

10.1186/s13578-016-0098-2 Assessment of IL-15Rα expression. (a) Blood samples in WT B6 or irradiated B6 mice (n = 4) at day 5 post T-cells transfer were stained with OVA-tetramer, anti-CD8 Ab and anti-IL-15Rα Ab, and analyzed by flow cytometry. OVA-tetramer and CD8 double positive T-cells were gated for further assessing the expression of IL-15Rα (solid lines). Gray shaded histograms represent isotype Ab controls. ***p* < 0.01. (b) Naïve OT-I CD8^+^ cells were activated in complete medium containing OVA I peptide and IL-2 for 3 days, followed by co-culturing with or without irradiated (10,000 rads) bone marrow-derived DCs for another 16 h. The resulting cells were then stained with anti-CD8 Ab and anti-IL-15Rα Ab, and analyzed by flow cytometry. CD8 positive T-cells were gated for further assessing the expression of IL-15Rα. **p* < 0.05. One representative experiment of three is shown.

## Abbreviations

Ab: antibody
AICD: activation-induced cell death
Atg7: autophagy-related gene-7
FITC: fluorescein isothiocyanate
FoxO: fork head-box family of transcription factor
Eomes: eomesodermin
IL: interleukin
KO: knockout
OVA: ovalbumin
PE: phycoerythrin
STAT5: signal transducers and activators of transcription-5
TCR: T-cell receptor
Te: effector T
Tm: memory T
TMRM: tetramethylrhodamine ester
ULK1: Unc-51-like kinase-1
Bcl2: B-cell leukemia/lymphoma-2
