# Supplementary figures and images for: IL-15 signaling promotes adoptive effector T-cell survival and memory formation in irradiation-induced lymphopenia
Source: Cell Biosci. 2016 May 6;6:30. doi: 10.1186/s13578-016-0098-2 (PMC4858849; doi:10.1186/s13578-016-0098-2)

## Supplementary Figure 1

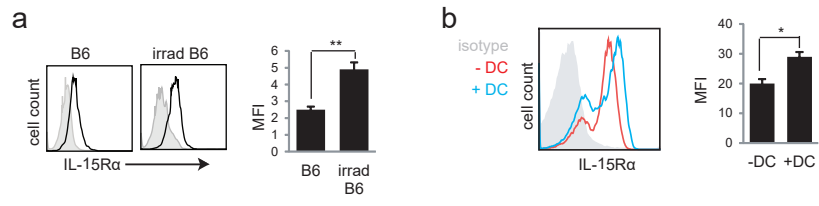

Supplement: Supplementary file 1 — 10.1186/s13578-016-0098-2 Assessment of IL-15Rα expression. (a) Blood samples in WT B6 or irradiated B6 mice (n = 4) at day 5 post T-cells transfer were stained with OVA-tetramer, anti-CD8 Ab and anti-IL-15Rα Ab, and analyzed by flow cytometry. OVA-tetramer and CD8 double positive T-cells were gated for further assessing the expression of IL-15Rα (solid lines). Gray shaded histograms represent isotype Ab controls. **p < 0.01. (b) Naïve OT-I CD8+ cells were activated in complete medium containing OVA I peptide and IL-2 for 3 days, followed by co-culturing with or without irradiated (10,000 rads) bone marrow-derived DCs for another 16 h. The resulting cells were then stained with anti-CD8 Ab and anti-IL-15Rα Ab, and analyzed by flow cytometry. CD8 positive T-cells were gated for further assessing the expression of IL-15Rα. *p < 0.05. One representative experiment of three is shown. [file 13578_2016_98_MOESM1_ESM.pdf]
